# Are You Happy? A Validation Study of a Tool Measuring Happiness

**DOI:** 10.3390/bs12080295

**Published:** 2022-08-19

**Authors:** Matteo Rizzato, Cinzia Di Dio, Laura Miraglia, Carlo Sam, Sharon D’Anzi, Michele Antonelli, Davide Donelli

**Affiliations:** 1 Humandive, c/o M. R. Coaching Sas, 33170 Pordenone, Italy; 2 Department of Psychology, Università Cattolica del Sacro Cuore, 20123 Milan, Italy; 3 Department of Public Health, AUSL-IRCCS Reggio Emilia, 42121 Reggio Emilia, Italy; 4 Cardiology Unit, Department of Cardiothoracic and Vascular Diseases, University Hospital, 43126 Parma, Italy

**Keywords:** happiness, measure, well-being, dynamic

## Abstract

This study aims at evaluating the psychometric properties of a new scale to measure experienced happiness—the Measure of Happiness (MH)—in a nonclinical sample composed of Italian adults from the general population. The MH was developed not only to provide a global measure of happiness, but also and more importantly to identify the specific areas of the individual’s life that are related to the experienced happiness. A total of 787 adults filled the MH and other self-report questionnaires, in order to assess the factor structure, reliability and external validity of the measure. The factorial analysis identified the following five dimensions: Psychophysics Status, Financial Status, Relational Private Sphere, Socio-Relational Sphere, and Life Perspective. The scale so defined was administered to a second independent group of 421 participants for the (multigroup) confirmatory factor analysis (CFA). A multigroup factor analysis based on gender confirmed the MH structure. The convergent validity of the MH was assessed by comparing the MH scores with a previously validated test of happiness and quality of life, as well as with dispositional constructs with which happiness is known to be negatively correlated, namely, anxiety and depression. The MH showed satisfactory psychometric properties and a strong significant positive relationship between the two measures of happiness, and a substantial negative association with the measures of anxiety and depression, supporting the validity of the MH to assess the construct of experienced happiness. The implications and possible applications of the MH are then discussed.

## 1. Introduction

One of the most important human dispositions is that of happiness. Happiness is a construct that outlines a positive feeling in the beholder and, more generally, well-being. It can be regarded as a relatively stable condition associated with various aspects of the individual’s life, and it differentiates from emotions like joy or sadness that, on the other hand, are transitory in nature. Understanding of happiness looms large in philosophical, psychological, scholarly, narrative and also governmental considerations, and still a definite consensus on what makes people truly happy has not been reached yet. Whenever asked, people should be able to generally tell whether they are happy or not [1]. However, if asked why they are happy, responses to this more specific question may be elusive or very general. As a matter of fact, the factors that may contribute to one’s happiness are varied. People may find happiness in wealth (economic forces [2]), activity levels [3], love, spirituality, and family conditions [4,5]. If happiness is more of a subjective attribute, so as it appears to be, then any efforts to measure happiness in a precise and objective manner may appear futile. Nevertheless, several attempts have been made over the past decades to quantify the level of experienced, subjective happiness and well-being. Over the years, the study of well-being and happiness, in fact, has increasingly become a field of great interest and importance [6,7,8]. One of the former and widely used measures of happiness is Bradburn’s [9] Affect Balance Scale, which assesses the balance of positive and negative affect experienced during the past four weeks, generally addressing the affective component associated with happiness. Similarly, the commonly used Positive and Negative Affect Scale (PANAS [10]) describes affective-emotional aspects of well-being and is comprised of positive and negative terms that are reported as distinct and independent concepts. From a cognitive standpoint, the Satisfaction With Life Scale [11] and the single-item Delighted-Terrible Scale [12] measure subjective well-being in terms of life satisfaction. Through these and other measures of well-being, respondents are usually asked to rate their level of positive and negative affect over a particular period of time or to make a judgment of their overall life quality, addressing either the affective or the cognitive dimensions associated with happiness.

Most recently, research on happiness has specifically moved into its temporal dimension. Some suggest that happiness can be transient, made of single ecstatic moments, when personal goals are achieved, for instance [13]; whereas others suggest that happiness can be a personal disposition and, as such, more durable in time [14]. Addressing fluctuating happiness and authentic-durable happiness, Dambrun and colleagues [15] designed and validated two scales of happiness: the Subjective Fluctuating Happiness Scale (SFHS) and the Subjective Authentic–Durable Happiness Scale (SA–DHS). The items of these two scales load on distinct factors, suggesting that fluctuating happiness and durable happiness are different dimensions of happiness. While both scales are robustly related to optimism, a sense of coherence, perceived resiliency and the presence of the search for meaning in life, the SA-DHS has proved to be more closely related to positive affectivity and life satisfaction, whereas the SFSH seems to be more linked to emotional negativity than to emotional positivity.

An attempt to measure the global “subjective happiness” was made by Lyubomirsky and Lepper [16], who developed a 4-item Subjective Happiness Scale. Though very brief, this scale has proved to be a reliable measure of global subjective happiness, as shown by positive correlations with other validated measures of happiness and well-being. This scale was developed and validated in 14 studies, and data was collected at different times and locations from students and older adults. The Subjective Happiness Scale addresses the construct of happiness by asking individuals to explicitly assess their level of happiness on a seven-point Likert scale. Two items require respondents to characterize themselves using both absolute ratings and ratings relative to peers; the other two items offer brief descriptions of happy and unhappy individuals and ask respondents the extent to which each characterization describes them. The Subjective Happiness Scale appears to fully satisfy the validation criteria that make it a reliable scale of global subjective happiness. In addition to the several scales developed to mainly measure adaptive characteristics of happiness and well-being, recent research has approached happiness from a different point of view, considering its maladaptive aspect, in which the evaluation of happiness or the fear of it is addressed as harmful to subjective and psychological well-being, further widening the understanding of happiness. Within this context, the Valuing Happiness Scale [17], the Fear of Happiness Scale [18] and the most recent Irrational Happiness Beliefs Scale (IHB [19]) have been developed to measure the degree of happiness aversion and the maladaptive aspect of it.

What lacks in the aforementioned measures of happiness and well-being is a specific reference to the source or sources of experienced happiness. Why are people happy? What specific aspects of their life make them more or less happy? As a matter of fact, happiness is a multifaceted construct that encompasses several dimensions of people’s lives, which may either contribute to increasing or decreasing the level of perceived happiness and well-being. Furthermore, profiling of happiness and well-being from the scales described above mainly derives from questions directly asking for the respondent’s affective and/or cognitive state in specific time periods or specific contexts. None of these measures, however, provide information on what particular dimensions of the respondent’s life may contribute to the global level of experienced happiness, failing to quantify the specific weight of these dimensions in the development of happiness.

This work aims at introducing a newly developed measure that makes an attempt to go beyond the mere global assessment of subjective happiness, but also and most importantly to identify the aspects of life that contribute critically to such a level. The central focus of the present study is to look at the dimensions that may influence happiness and well-being as the sphere of affect, perceived economic status, and health. Therefore, the present research aims to respond to the need of enhancing the framework of evaluation methods about this important construct, by proposing a new self-report questionnaire for the assessment of happiness and its components. For all these reasons, the current study aims to present a new measure for the assessment of happiness, the Measure of Happiness (MH), based on an integrated perspective and inspired by the criteria of brevity, good psychometric properties, and usefulness for research activity and for governments, national and international associations, or social institutions that might be motivated to identify the reasons behind the level of happiness of individuals or communities. For this purpose, we first assessed the structure of the MH, with an exploratory and a (multigroup) confirmatory factor analysis; we hypothesized that the MH would have at least a four factor-structure that emphasized the dimensions of people’s lives, i.e., perceived physical and mental health status, economic conditions, family and social relations, and future perspective. Additionally, the reliability and external (convergent and divergent) validity were examined. The convergent validity was assessed by correlating the MH scores with those of the Subjective Happiness Scale [16] described above, which has been proven to be a highly reliable and valid measure of subjective happiness, and the WHOQOL-BREF (Italian version: De Girolamo et al. 2000 [20]), which proved to be valid and highly reliable in a large international field trial, measuring the quality of life perception. Additionally, validation of the MH was carried out by comparing scores with two widely used scales assessing anxiety and depression, namely, the State-Trait Anxiety Inventory (STAI [21,22,23]) and the Beck Depression Inventory (BDI [24]). The MH capability to measure happiness would be validated if it is positively associated with the Subjective Happiness Scale and the WHOQOL and negatively associated with the scales of anxiety and depression.

## 2. Materials and Methods

### 2.1. Participants

Data were collected from two samples. The first sample was employed for exploratory factor analysis and internal consistency. The second sample was used for confirmatory factor analysis and external validity. Participants in Sample 1 consisted of three hundred and sixty-six (366) Italian adult (50.3% women; mean age = 49.5, *SD* = 1.24; range 22–84 years; 49.7% men, mean age = 49.1, *SD* = 1.27; range 20–80 years). Most participants reside in northern Italy (81.7%), 7.4% in central Italy, 8.7% in southern Italy, and the remaining 2.2% in Sicily and Sardinia. Few participants reported the use of drugs (0.5%) or psychotropic drugs (3%). The sociodemographic characteristics of Sample 1 are reported in Table 1.

Sample 2 included four hundred forty-nine (421) Italian adults (53.7% women; mean age = 42.19, *SD* = 10.2; range 22–66 years; 46.3% men, mean age = 45.05, *SD* = 10.85; range 22–72 years). As with the first sample, most participants live in northern Italy (60.6%), 18.5% reside in central Italy, 13.3% in the south, and the remainder in Sicily and Sardinia (7.6%). Overall, 3.1% reported using drugs, and 1% using psychotropic drugs. The sociodemographic characteristics of Sample 2 are reported in Table 1.

The only inclusion criterion for participating in data collection was to be of legal age, that is, over 18 years old.

### 2.2. Measures

#### 2.2.1. The Measure of Happiness (MH)

The Measure of Happiness (MH) was developed to assess the construct of happiness through questions addressing several aspects of the individual’s life. The content of the items was implemented by reflecting on the events that are most commonly associated with the level of the individual’s well-being, as introduced above. In general, the questionnaire aims to evaluate various dimensions of the subject’s everyday life, such as the individual within the domestic and relational contexts. The MH initial version was created to reflect the core aspects of the construct and consisted of 32 items grouped into four macro-areas of investigation: perceived health condition (psychological and physical wellbeing), economic situation (the subject’s financial status), family/social relations (the personal network of relationships, including those with family members, friends, and colleagues), and future perspective (self-development, long-term goals, and life changes).

After a preliminary analysis aimed at improving the questionnaire’s internal consistency, the number of items was eventually reduced from 32 to 14, and the number of domains was increased from four to five: in particular, the macro-area about family/social relationships was split into two domains (one about private and family relationships and the other one about public and professional relationships), whereas the other three macro-areas were converted into three corresponding domains. The five domains were the following: Psychophysics Status (3 items), which investigates the subject’s psychophysical health; the Financial Status (3 items), which describes the subject’s economic condition; the Relational Private Sphere (3 items) and Socio-Relational Sphere (2 items), which explore the network of different personal relationships; and the Life Perspective (3 items), which includes long-term goals, life changes, and attitudes towards self-development.

Here, we report a full list of the 14 items selected for the final version of the MH: each question is reported in Italian and accompanied by an English translation for better comprehensiveness and possible validation studies in English speaking countries.

- Psychophysics Status:
  ○ *Come valuti il rapporto con il tuo corpo?*
    ▪ How do you evaluate your relationship with your body?
  ○ *Come valuti il tuo livello di equilibrio mentale e fisico?*
    ▪ How do you evaluate your level of mental and physical balance?
  ○ *Come valuti il tuo rapporto con te stesso?*
    ▪ How do you evaluate your relationship with yourself?
- Financial Status:
  ○ *Quanto ritieni di essere realizzato in questo momento?*
    ▪ How fulfilled do you feel with your life at this moment?
  ○ *Quanto sei soddisfatto della tua condizione finanziaria?*
    ▪ How satisfied are you with your financial situation?
  ○ *Quanto ti senti solido finanziariamente?*
    ▪ How financially sound do you feel?
- Relational Private Sphere:
  ○ *Come valuti la qualità dei tuoi rapporti con i tuoi affetti principali?*
    ▪ How do you evaluate the quality of your relationships with your dear ones?
  ○ *Quanto ti soddisfa l’atmosfera che si vive nella tua attuale casa?*
    ▪ At present, how satisfied are you with the atmosphere in your home?
  ○ *Secondo te, i membri della tua famiglia, quanto ti stimano?*
    ▪ In your opinion, how much do your family members appreciate you?
- Socio-Relational Sphere:
  ○ *Quanto pensi che le persone, in generale, siano felici di relazionarsi con te?*
    ▪ In general, how happy do you think people are to interact with you?
  ○ *Quanto ritieni apprezzati i tuoi comportamenti nella società?*
    ▪ How much do you think your behavior is appreciated in society?
- Life Perspective:
  ○ *Quanto ritieni importante porti degli obiettivi di lungo termine?*
    ▪ How important is it to you to set long-term goals?
  ○ *Quanto ti interessi al tuo miglioramento personale?*
    ▪ How much are you engaged in self-improvement?
  ○ *Quanto ti senti flessibile di fronte ai cambiamenti della vita?*
    ▪ How adaptable do you feel to major changes in your life?

The MH is a fixed measure: Each item is rated on a 10-point Likert scale ranging from 1 (not at all) to 10 (very, very much). The scores obtained for every single item composing the various domains are used for the computation of the happiness index for that specific domain. Responses to the 14 items were summed to provide the domains and the total scores. The scores for each domain are then added up to calculate the total level of experienced happiness. The scores obtained from each question contribute to the final happiness score ranging from 0—complete lack of happiness—to 100—totally happy.

#### 2.2.2. Subjective Happiness Scale (SHS)

The Subjective Happiness Scale (SHS) was developed by Lyubomirsky and Lepper [16]. This scale was selected and preferred over others because of methodological issues that make SHS a valid and reliable measure of happiness. For details on the validation procedure, please refer to the original paper. In brief, the SHS is a 4-item scale of subjective happiness that was derived from an original pool of 13 self-report items. From these original items, six were discarded from further testing based upon high semantic similarity. An additional three items were discarded because they did not load onto a single interpretable factor in a principal component analysis performed on the items. Responses are recorded on a 7-point Likert scale. Higher scores reflect greater happiness. The validity of the test was assessed by comparing scores on the SHS with five other measures of happiness and well-being. More specifically, the SHS was validated against the Affect Balance Scale [9], the Delighted-Terrible Scale [12], the Global Happiness Item [9], the Recent Happiness Item [25], and the Satisfaction With Life Scale [11]. The scores of the 4 items were summed. Cronbach’s α was 0.18.

#### 2.2.3. State–Trait Anxiety Inventory (STAI)—Anxiety

The State–Trait Anxiety Inventory (STAI) is an introspective psychological inventory constructed by Charles Spielberger and colleagues [21,22,23] based on the state–trait distinction proposed by Cattell [26]. The STAI is a 40-item questionnaire consisting of two sections, one assessing anxiety of “state” (Y1, 20 items) and the other one assessing anxiety of “trait” (Y2, 20 items). The anxiety of “state” indicates how much an individual perceives him/herself as anxious in that specific moment, namely, when performing the test; the anxiety of “trait” refers to how a person usually feels, independently of the current emotion-state. The responses are collected on a 4-point-Likert scale ranging from 1 (not at all) to 4 (very much). Some examples of the questionnaire items are the following: “I feel calm”, “I feel secure”, “I feel tensed”, and “I have regrets”. The total score is obtained by summing all scores and being careful to reverse the scores for the positive items (the former two questions in the present example). The total score for each session ranges between 20 and 80. Higher scores indicate greater anxiety. Scores above 60 index severe anxiety. Scoring was obtained by summing up the items belonging to the two scales. Cronbach’s α for STAY-1 was 0.94, while Cronbach’s α for STAY-2 was 0.95.

#### 2.2.4. Beck Depression Inventory (BDI)

The Beck Depression Inventory (BDI [24]) is a 21-item questionnaire measuring cognitive, motivational, affective, and behavioral symptoms of mild depression or dysphoria, rated for the past week. The items are represented by self-assessments of the respondent’s emotion/affective state and physical condition. The respondent is given a general instruction to indicate the answer, among four possibilities, that gets the closest to what the subject thinks or feels. For example, assessing an emotion-state: “I don’t feel sad; I feel sad; I feel sad all the time and I cannot feel any better; I feel so sad and unhappy that I cannot bear it”; assessing a physical condition: “I have not lost much weight lately; I have lost more than two and a half kilos; I have lost more than five kilos; I have lost more than seven and a half kilos”. All the answers are ordered numerically from 0 to 3, where 3 always reflects the worst possible condition. The total score is therefore comprised between 0 and 63. The higher scores index greater depression. Scores above 29 indicate severe depression. The scoring was obtained by summing the scores of each item. Cronbach’s α was 0.89.

#### 2.2.5. WHOQOL-BREF

The WHOQOL-BREF is an abbreviated version of the WHOQOL-100 self-report questionnaire [27], which had demonstrated clear validity and reliability in a large international field trial, measuring the perception of the quality of life. The questionnaire has been translated into many languages and validated in various countries including Italy [20]. It is composed of 26 items, distributed into four domains: physical health, psychological, social relationships, and the environment. Each domain considers several aspects: (a) physical domain: pain, discomfort, energy, fatigue, sleep, rest; (b) psychological domain: feelings, appearance self-esteem, memory, and concentration; (c) social relations domain: social relations, social support, sexual activity; and (d) environment domain: safety, home environment, finances, leisure, transport, social care. Four types of 5-point Likert scales distributed among the domains were used in the WHOQOL-BREF. Items inquire “how much”, “how completely”, “how often”, “how good” or “how satisfied” the respondent felt in the last two weeks; different response scales are distributed across the domains [27]. The scoring was calculated by averaging the items for each factor. Cronbach’s α was 0.91.

### 2.3. Procedure

The questionnaires were administered through a custom platform designed for this study. The participants received an email with the link to the online registration form, following which they entered the personal dashboard with the questionnaires to complete. The platform implements a custom algorithm that automatically changes the way each questionnaire is administered to the user by randomly changing the sequence of the questions and the order of the list of answers, thus avoiding any iterative behavior. Once the survey is submitted, the user can start a new questionnaire from the list of the remaining available questionnaires or log out from the platform and resume it in a second step within two hours. Data are sent safely through a Secure Socket Layer (SSL), i.e., the standard security technology to establish an encrypted link between a web server and a browser, which ensures that all data are kept reserved and integral. In addition to providing a link to the survey, participants were presented with all the necessary information, including the purpose of the study, the instructions, and the duration of the survey. They were also informed that they could leave the session any time and resume the test for completion in a second step within a time frame of two hours. This was done to make sure that the respondents completed the whole test and still that all data were collected at approximately the same time point.

The questionnaires were completed online through a guided procedure that began with an information page, notifying the respondents about the general aim of the study and the procedural terms. The information page was followed by the consent page that the respondent had to read and approve before having access to the actual experimental session. Only those who gave their online informed consent were included in the data collection. Furthermore, all participants were treated in accordance with the ethical guidelines for research provided by the Declaration of Helsinki and its revisions and the study was approved by the local ethic committee (Università Cattolica del Sacro Cuore, Milan). After recording the respondents’ formal consent to participate in the study, demographic data were collected. The experimental session of the first study began with the administration of the full-set MH questionnaire. In the second study, participants completed the refined 14-items version of the MH. Some sociodemographic data were collected before the administration of questionnaires. In order to test the validity of the scale, in both sessions other four questionnaires concerning happiness, anxiety and depression were included and they were presented in a random order across respondents. The items of the questionnaires were administered singularly. After providing a response, the participant was led to the following question or statement. Each questionnaire was introduced by specific instructions explaining to the participant the response criteria and their meaning (as it is specified in the validated version of the questionnaire). For example, for the anxiety questionnaire the respondent was explained that he or she would have needed to answer to each statement on a scale ranging 0 (totally disagree) to 4 (totally agree). The whole test ended at completion of the fifth questionnaire.

### 2.4. Data Analysis

Data analyses were performed using SPSS Version 27.0 and Jamovi statistical software (Version 2.2) [Computer Software]. The analyses were carried out on two independent samples to overcome the problem of common error variance, which could occur if the same participants are repeatedly involved in the procedure. To enhance the reliability of the scale, the ideal procedure is then to develop the scale on sample A and then test it on an independent sample B [28,29]. Descriptive statistics were calculated to assess normal distribution. Adequacy of the sample was investigated with Bartlett’s test of sphericity (*p* < 0.05) and Kaiser-Meyer-Olkin (KMO > 0.70) test. Hull’s method suggests considering only items with a loading >0.40 on a single factor for further analysis. Cronbach’s alpha coefficient was computed for the reliability statistic. First, A Principal Component Analysis (PCA) was carried out to examine the underlying factor structure of the MH, using the data from Sample 1. Oblique rotation (*direct oblimin*) was used because the factors were presumably related to each other rather than independent. Delta was set to 0. Then, we investigated the structure with Cattell’s Scree test [30] and Horn’s Parallel Analysis [31] techniques. We expected a 4-factor model highlighting the different macro-areas that make up the scale, namely perceived health condition, economic situation, family/social relations, and future perspective.

The factorial validity of the MH was assessed with confirmatory factor analysis (CFA) using the data from Sample 2. To determine the fit of the CFA model, according to Hu and Bentler’s guidelines [32], we considered the χ2 test statistic, the comparative fit index (CFI), the Tucker-Lewis index (TLI), the Root Mean Square Error of Approximation (RMSEA), and the Standardized Root Mean Square Residual (SRMR). Generally, CFI and TLI values larger than 0.90 are taken to indicate acceptable fit [32,33,34], although values greater than 0.95 are desirable [35]. RMSEA values lower than 0.05 indicate close fit [36], values between 0.05 and 0.08 indicate acceptable fit, values between 0.08 and 0.10 indicate mediocre fit, and values greater than 0.10 indicate poor fit [37]. SRMR values range from 0 to 1.0, with well-fitting models obtaining values smaller than 0.05 [38]; however, values as high as 0.08 are deemed acceptable [32,39].

Factorial invariance was tested by comparing a series of increasingly restrictive models using the software Jasp (version 0.14; JASP Team 2020) with progressively more restrictive hypotheses about equality across gender groups [40]. For this purpose, we used the changes in CFI and RMSEA [41,42]. Specifically, a ΔCFI less than or equal to 0.01 and a ΔRMSEA less than or equal to 0.015 between the most restrictive model and the previous one indicates that the hypothesis of between-group invariance should not be rejected.

The convergent and discriminant validity of the MH scale was assessed by computing Pearson correlations between the MH factors and related constructs, i.e., quality of life, happiness, anxiety, and depression.

## 3. Results

### 3.1. Happiness Scales: Descriptive Analysis and Internal Consistency

Descriptive analysis for all the scales is summarized in Table 2. Skewness and kurtosis of the MH total score were between −1 and +1 (−0.39 and 0.11, respectively) and the mean value of the total score was 104.84 (*SD* = 14.87).

We assessed the internal consistency reliability for the items on the MH using Cronbach’s alpha coefficient. Cronbach’s alpha is fairly high for the proposed MH (α = 0.85). However, the elimination of two items suggested an improvement, yielding a Cronbach’s alpha coefficient of 0.89. The initial item pool was reduced to 16 items.

To assess any effects on MH scores as functions of age and gender, we correlated these variables with the MH factors. No significant correlations were found, indicating that the scores were not influenced by participants’ gender and age.

### 3.2. MH Structure: Exploratory Factor Analysis

Kaiser–Meyer–Olkin (KMO) and Bartlett’s test of sphericity were conducted to explore the suitability of the data for the factor analysis. The KMO value was greater than 0.60 (KMO = 0.86), and Bartlett’s test of sphericity was statistically significant.

**Figure 1:**
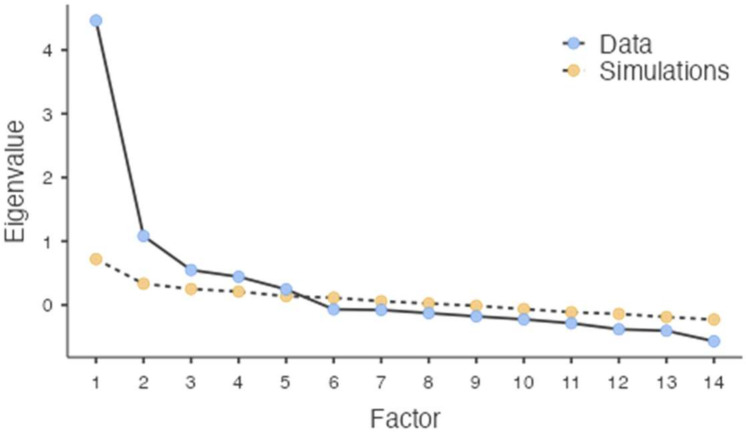
Scree plot.

We employed three techniques to identify the number of factors to retain: Kaiser’s eigenvalue greater than one technique [43], Cattell’s Scree test [30], and Horn’s Parallel Analysis [31]. Therefore, the eigenvalue method and scree plot were firstly taken into account for the factors to be extracted. Principal component analysis (PCA) with an oblique rotation (*oblimin*) was performed, and a four-factor structure was first determined based on the eigenvalue method, thus explaining 65.35% of the total variance. However, the scree plot suggested extracting five factors (Figure 1). Secondly, parallel analysis was applied to confirm the underlying latent factor structure. Parallel analysis is defined as the most accurate method for component extraction [44,45] compared to the aforementioned techniques. For a factor to be retained, the number of eigenvalues of the factors retained from the sample data must be greater than the eigenvalues of the random data. The results of the analysis showed five components with eigenvalues exceeding the corresponding criterion values for a randomly generated data matrix of the same size. The first five eigenvalues (4.46, 1.08, 0.55, 0.44, and 0.25) were notably lower than the corresponding ones from the PCA (5.11, 1.79, 1.29, 1.05, and 0.99). Thus, the parallel analysis suggested a five-component solution as the most fitting—with 73.05% of the variability explained—confirming the result of the scree plot.

Furthermore, the pool of 16 items was reduced to 14: two items had low rotated charges and loaded on more than one factor. Individual item loadings on the retained components are listed in Table 3. The item loadings on the scale were robust based on Hair et al.’s [46] notion in which the criterion of the inclusion of the item on factor loading should be above 0.3. The first component, explaining 36.48% of the variance, had three significant loadings (rotated loadings between 0.78 and 0.88), allowing it to be identified as Psychophysics Status (MH-PS). The second component, explaining 12.77% of the variance, was determined by three items as well (rotated loadings between 0.54 and 0.96), concerning Financial Status (MH-FS). The third, explaining 9.19% of the variance, was labelled Relational Private Sphere (MH-RPS), as the three items (rotated loadings between 0.54 and 0.96) loading onto this component refer to family members and the home environment. The fourth component was composed of two items (rotated loadings of 0.87 and 0.90), explaining 7.52% of the variance, was labelled Socio-Relational Sphere (MH-SRS) and differs from the previous one because it assesses the relationship with members of the society. Finally, tree items were loaded on the fifth component (rotated loadings between 0.38 and 0.88), explaining 7.09% of the variance, which was identified as Life Perspective (MH-LP) as it investigates future prospects and long-term goals.

### 3.3. CFA of the Five-Factor Solution and Structural Invariance

**Figure 2:**
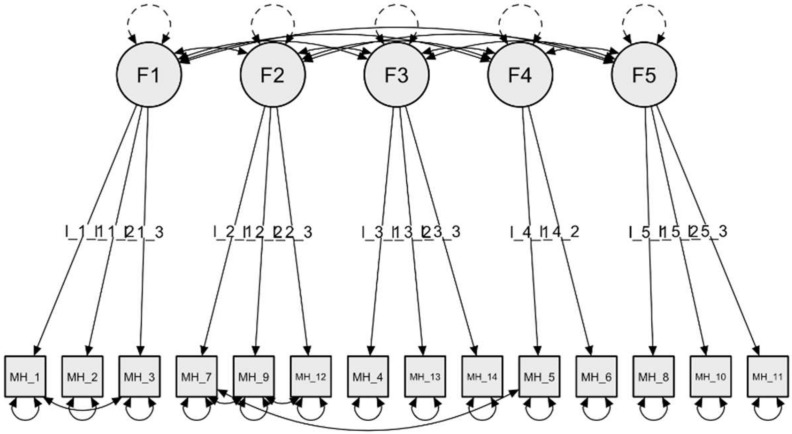
Graphical summary of the CFA obtained from the 14-item Measure of Happiness (MH).

To investigate the reliability of these factors, the MH items were subjected to a CFA using the data from the second sample (N = 431). The goodness-of-fit indices indicated a satisfactory fit of the five-factor model. Although most of the indices had reached the recommended cut-off values (CFI = 0.93; TLI = 0.91; SRMR = 0.07), not all of them met the values needed to consider the model acceptable (RMSEA = 0.097, 90% confidence interval [CI] = 0.09–0.11). Careful inspection of modification indices indicated that the model could improve if four pairs of items were correlated. The new CFA showed better fit indices as follows: CFI = 0.98; TLI = 0.97; SRMR = 0.03; RMSEA = 0.06 (90% confidence interval [CI] = 0.04–0.07). The Model Chi-Square was significant (χ2/df = 2.37, *p* < 0.001) (Figure 2).

Next, to investigate the efficacy of the model across gender, separate multi-group CFAs were carried out for women (N = 226) and men (N = 195). The CFA on the refined and fully unconstrained model indicated adequate fit, χ2 = 278.38; df = 122; CFI = 0.96, TLI = 0.94; SRMR = 0.04; RMSEA = 0.08 (CI = 0.07–0.09), suggesting factorial invariance across gender. Metric invariance (χ2 = 289.61; df = 131; CFI = 0.96, TLI = 0.95; SRMR = 0.05; RMSEA = 0.08) and scalar invariance (χ2 = 297.27; df = 140; CFI = 0.96, TLI = 0.95; SRMR = 0.05; RMSEA = 0.07) were obtained, suggesting that a constrained model fit the data best. The assumption of invariance is accepted since the difference of CFI and RMSEA indices between the most restrictive model and the previous one is smaller than 0.1 and 0.015, respectively.

### 3.4. Convergent and Discriminant Validity

To assess convergent validity, the MH was correlated with the Subjective Happiness Scale (SHS) and WHOQOL. The SHS and the WHOQOL were selected as the term of comparison for validation since they have shown high validity and reliability in assessing the subjective level of the individual’s happiness and quality of life, as fully described above. The Pearson’s correlation between the MH and the Subjective Happiness Scale was revealed to be substantial: all MH factors correlated significantly, and in the hypothesized direction, with the SHS (with *r* ranging between |0.30| and |0.46|, *p* < 0.001). The Psychophysics Status of the MH generally positively correlated with the dimensions of the WHOQOL, except for the *Environment domain*, *r* = 0.21, *p* < 0.05; *r* = 0.26, *p* < 0.01, *r* = 0.20, *p* < 0.05. No other correlations were found.

Additionally, discriminant validity was assessed by comparing scores obtained from the MH with scores obtained from two dispositional constructs that are opposite to that of happiness, namely, anxiety (STAI-1, STAI-2) and depression (BDI). MH factors correlated significantly and negatively with both STAI-1 (with *r* ranging between |−0.28| and |−0.51|, *p* < 0.001), STAI-2 (with *r* ranging between |−0.27| and |−0.44|, *p* < 0.001), and BDI (with *r* ranging between |−0.22| and |−0.55|, *p* < 0.001). The results showed that individuals who scored high on the MH happiness scale were low on measures of anxiety and depression. The correlation statistic is summarized in Table 4.

## 4. Discussion

The aim of the study was to evaluate the psychometric properties of the Measure of Happiness (MH) in an Italian sample. The scale was designed to meet the criteria of brevity, good psychometric properties and usefulness for research activity and a wide range of application as discussed below. To this purpose, we first tested the factorial structure and reliability of the scale, then its external validity by examining the associations with MH, happiness, quality of life, anxiety, and depression. The MH showed satisfactory psychometric properties, with a clear and theoretically relevant factor structure, adequate internal consistency, and excellent construct validity. The MH has been developed as a multidimensional scale, with each item evaluating objective aspects of the respondents’ life as well as, eventually, the related level of satisfaction. As a matter of fact, happiness is not a monolithic construct; rather, its realization depends on a number of dimensions of people’s lives, such as wealth [2], activity levels [3], relationships and family conditions [4,5], which can lead to an increase or decrease in its perception. Additionally, the definition of the factors associated with experienced happiness, which are tied to conditions that characterize the quality of daily life over a fairly prolonged period of time, lends support to the idea that happiness as measured by the MH captures a quite stable rather than transient condition [14]. As a reflection of this, the factorial analysis indicates a five-factor structure, also confirmed by the confirmatory factor analysis (CFA). The extracted factors reflect the core aspects of people’s lives: the Psychophysics Status (F1) investigates the health of the respondent by questioning the psychophysics status; the Financial Status (F2) deepens the economic condition making questions about the financial status; the Relational Private Sphere (F3) and Socio-Relational Sphere (F4) explore the private and social sphere, respectively; and finally, the Life Perspective (F5) investigates future plans and long-term goals. The model demonstrated metric and scalar invariance across gender. This means that women and men interpret the items in the same way and that the factor loadings are stable across groups.

Our data provided support to the validity of the MH to assess the construct of experienced happiness: positive correlations were found comparing the MH scores with scores for happiness and quality of life obtained through the Subjective Happiness Scale (SHS; [16]) and the WHOQOL ([27]; Italian version [20]). Additionally, our results also supported satisfactory discriminant validity with measures of anxiety (STAI [21,22,23]) and depression (BDI [24]), revealing that individuals who scored high on the MH happiness scale were low on measures of anxiety and depression.

A review of the happiness literature reveals that most existing happiness measures attempt to assess happiness either globally or in relation to a specific level of happiness or subjective well-being. As already described, the MH tests for the construct of happiness by assessing several dimensions of the individual’s daily life, thus providing both an overall score for happiness and independent indexes of satisfaction related to the tested dimensions. Therefore, the novelty of this scale, with respect to already existing measures of happiness (see, for example, [9,11,12,15,47,48]), stems from its multidimensionality. The innovative aspect of MH is that it explicitly articulates the dimensions of the individual’s life that contribute to the overall level of experienced happiness, thus succeeding in quantifying the specific weight of these dimensions in the development of happiness. Not by chance, the first factor reflecting the level of happiness is psychophysical well-being. Mental and physical health are extremely relevant in the experience of well-being and their lack results in an unbalance towards states of depression and anxiety, which are negatively associated with happiness. For example, many perceived stress and depressive disorders at work may be caused by poor mental and physical health [49,50]. On the contrary, physical activity is associated with health and general wellbeing and proved to be an effective coping strategy contrasting stress [51]. Additionally, studies evaluating the psycho-physiological parameters associated with physical activity have demonstrated its protective effects on coronary heart disease [52] and depression [53,54]. Similarly, an individual’s socio-economic status has been proved to contribute substantially to perceived well-being and happiness, as evidenced by several studies indicating economic-financial wellness as one of the predominant contributors to personal well-being [55,56]. Likewise, people’s relational asset is of paramount importance in perceived happiness [57]. Human beings are made for relationships, as demonstrated by the negative response of our system when these are not functional, and by the positive/protective response of our system when these are of a good quality. When we are in a social context, where the relationship is active, our system releases oxytocin, a hormone whose discharge promotes relationships through a sense of affiliation, trust and motivation [58]. Positive relationships also show beneficial physiological effects through: (1) interaction with the cardiovascular system, measured in terms of heart rate during the day, evening, and sleep, as well as blood pressure during relationships and connections; (2) a stronger response of the immune system when under stress; and (3) influence on the hormonal release of oxytocin and cortisol by the neuroendocrine system [59]. On the opposite end, social isolation is often linked to depressive states [60].

A further strength of the MH is that it addresses the construct of happiness by Evaluating tangible life dimensions rather than asking people idiosyncratic evaluations. Existing validated tests on happiness address the construct with questions that directly ask for the respondent’s level of happiness and well-being by mostly using direct terminology tackling associated emotion/affective states (e.g., happiness, displeasure, beatitude, inner peace); on the other hand, the present scale assesses happiness indirectly, asking questions that require the respondents to give an evaluation of specific living conditions and, eventually, to indicate how much they recognize themselves in a specific circumstance or condition. The MH items never require the respondents to self-assess their emotional states. In this sense, the MH can be regarded as an objective measure of happiness. Hence, the structure of the MH offers the unique opportunity to identify the specific areas or dimensions of the respondent’s life that most contribute to his or her level of happiness or unhappiness. At the individual level, the usefulness of the questionnaire can be at least two-fold: on the one hand, the completion of the questionnaire itself may serve as a means of self-reflection on personal dimensions related to the individual’s level of life satisfaction that the subject had never really thought about. The questionnaire, in fact, “forces” the respondent to reflect on daily life issues, making them explicit, such as those related to the health and economic condition, family and social relations, and future perspective. In this respect, there is ample evidence about the effect of self-reflection on a number of psychological [61], clinical [62,63,64,65], educational [66,67], and work [68] practices.

Overall, as a development of previous theoretical frameworks and happiness scales, our study results demonstrate that the MH provides an objective measurement of human well-being by investigating the most relevant life aspects. In particular, the MH does not simply record positive or negative life aspects, but it underscores the quantitative and qualitative characteristics of five domains, well described in specific questions and based on a long-lasting time frame. In brief, the MH aims to highlight and quantify the specific “sources” of happiness, namely all life aspects that influence this construct the most, thus ultimately contributing to improve individual self-awareness.

Additionally, the MH may be used in specific professional settings, such as clinical ones, to aid the therapist to identify critical aspects of the patient’s life that require more attention during therapy. At a global level, the critical markers of happiness and well-being can be correlated with a variety of factors like, for example, the responders’ age, gender, religion, cultural background, political trends, education, etc., opening therefore to a wide range of possibilities in terms of application. The information gained thereafter could be used to favor and promote well-being at the community level. Most recently, some governments are making happiness their mission and commitment, through the implementation of policies, plans, projects, and services, to provide a nurturing environment for the happiness of individuals, families, and communities. Besides governments, also companies, education services, and care facilities could benefit from evaluating the level of individuals’ happiness by addressing critical areas of intervention to improve the quality of life within each respective area of competence, such as, broadly speaking, the work environment and the quality of services.

### Study Limitations

Our study has some limitations: first of all, the sample size was quite large, but only composed of Italian participants, namely people speaking Italian and living in Italy. Future research should focus on cross-cultural comparisons required to support the MH validity in cohorts of subjects with different nationalities and backgrounds. Secondly, our study only involved adult participants, but measuring happiness is important in children and adolescents too; in perspective, different scales, possibly visual analogue ones, with simplified items and adapted questionnaires should be used in these subjects to obtain reliable information. Thirdly, the MH was designed as a standard questionnaire; another option to be explored in future studies would be to implement a dynamic tree-like questionnaire, in which each response leads to a specific alternative pattern of questions, so that responders may be presented with different questions based on the answer given to previous items (answer piping).

## 5. Concluding Remarks

There is potential for happiness, but people are often unable to experience it. Happiness cannot be generally associated with single factors such as the national average income, socio-cultural and economic background, or even physical well-being. It is a multidimensional construct strictly related to everyone’s characteristics. The MH represents an objective quantitative and qualitative analysis of different aspects of the individual’s life that contribute to the global level of experienced happiness. This offers several advantages: through its completion, the individual is led to a reflection process on specific personal life issues, which may aid him or her to think about criticalities within, for example, the personal, affective, work, finance and physical domains. Identifying critical life issues that compromise an individual’s well-being and happiness can be the first step to address them effectively. This would be important, for instance, in clinical psychology and public health to improve individuals’ self-awareness and quality of life. For example, in the workplace, MH can be used to measure the level of well-being and life satisfaction of human resources and to identify the most critical issues that may undermine the employee’s happiness. On a more global level, this tool can be useful for different organizational, political and social settings and for different purposes, depending on the focus of attention as fully discussed.

## Figures and Tables

**Table 1:** Sociodemographic characteristics of samples.

Sociodemographic Characteristics	Sample 1 *N* = 366	Sample 2 *N* = 421
Age, mean ± SD	49.28 ± 16.93	43.48 ± 10.61
Gender	N (%)	N (%)
Male	182 (49.7%)	195 (46.3%)
Female	184 (50.3%)	226 (53.7%)
Residence	N (%)	N (%)
North Italy	299 (81.7%)	255 (60.6%)
Centre Italy	27 (7.4%)	78 (18.5%)
South Italy	32 (8.7%)	56 (13.3%)
Sicily and Sardinia	8 (2.2%)	32 (7.6%)
Educational level	N (%)	N (%)
Middle school or below	Nd	11 (2,6%)
High school	Nd	252 (59,9%)
Graduate school	Nd	158 (37,5%)
Mean annual income (euros)	N (%)	N (%)
<21,000	Nd	166 (39,4%)
21,000–60,000	Nd	214 (50,8%)
>60,000	Nd	41 (9,7%)
Drugs	N (%)	N (%)
Yes	2 (0.5%)	13 (3.1%)
No	364 (99.5%)	408 (96.9%)
Psychotropic drugs	N (%)	N (%)
Yes	11 (3.0%)	4 (1.0%)
No	355 (97.0%)	417 (99.0%)

**Table 2:** Descriptive analysis of the Measure of Happiness (MH).

Descriptive Statistics		Max.	Mean	SD	Skewness	Kurtosis
Scale	Min.					
MH_F1	3	30	22.14	4.9	−0.84	0.84
MH_F2	3	30	19.21	5.55	−0.52	−0.12
MH_F3	11	30	24.64	4.06	−0.93	0.74
MH_F4	8	20	15.14	2.28	−0.08	−0.01
MH_F5	7	30	23.70	4.093	−1.01	1.45
MH_TOT	53	140	104.84	14.87	−0.39	0.11

**Table 3:** Factor structure and reliability of Measure of Happiness (MH).

MH Items	Factor 1 Psychophysics Status	Factor 2 Financial Status	Factor 3 Relational Private Sphere	Factor 4 Socio-Relational Sphere	Factor 5 Life Perspective
1. Come valuti il rapporto con il tuo corpo?	0.869				
2. Come valuti il tuo livello di equilibrio mentale e fisico?	0.781				
3. Come valuti il tuo rapporto con te stesso?	0.883				
4. Quanto ritieni di essere realizzato in questo momento?		0.538			
5. Quanto sei soddisfatto della tua condizione finanziaria?		0.929			
6. Quanto ti senti solido finanziariamente?		0.959			
7. Come valuti la qualità dei tuoi rapporti con i tuoi affetti principali?			−0.829		
8. Quanto ti soddisfa l’atmosfera che si vive nella tua attuale casa?			−0.781		
9. Secondo te, i membri della tua famiglia, quanto ti stimano?			−0.718		
10. Quanto pensi che le persone, in generale, siano felici di relazionarsi con te?				0.869	
11. Quanto ritieni apprezzati i tuoi comportamenti nella società?				0.903	
12. Quanto ritieni importante porti degli obiettivi di lungo termine?					0.877
13. Quanto ti interessi al tuo miglioramento personale?					0.725
14. Quanto ti senti flessibile di fronte ai cambiamenti della vita?					0.389
% of explained variance	36.48%	12.77%	9.19%	7.52%	7.09%
Cronbach’s alpha	0.85	0.82	0.75	0.81	0.61

Note: Extraction method: Principal Component Analysis. Rotation method: Promax with Kaiser Normalization. a: Rotation converged in 7 iterations.

**Table 4:** Pearson’s correlations.

	Psychophysics Status	Financial Status	Relational Private Sphere	Socio-Relational Sphere	Life Perspective
SHS	0.46 **	0.43 **	0.30 **	0.35 **	0.34 **
WHO-F1	0.21 *	0.12	0.14	0.15	−0.03
WHO-F2	0.26 **	0.14	0.04	0.11	0.02
WHO-F3	0.20 *	0.15	0.10	0.17	−0.004
WHO-F4	0.11	0.13	0.03	0.18	0.05
STAI-1	−0.51 **	−0.44 **	−0.39 **	−0.35 **	−0.28 **
STAI-2	−0.44 **	−0.31 **	−0.33 **	−0.27 **	−0.28 **
BDI	−0.55 **	−0.31 **	−0.37 **	−0.22 **	−0.25 **

* Correlation is significant at the 0.05 level (2-tailed). ** Correlation is significant at the 0.01 level (2-tailed).

## Data Availability

The data presented in this study are available on request from the corresponding author. The data are not publicly available due to privacy reasons.
